# The Treatment of Ovarian and Uterine Tumors

**Published:** 1885-04

**Authors:** T. J. Word

**Affiliations:** Atlanta, Ga.


### THE TREATMENT OF OVARIAN AND UTERINE TUMORS.

BY T. J. WORD, M. D., ATLANTA, GA.

In presenting the history and treatment of the following cases, I deem it due, alike to myself and the profession, to give expression to my belief in the curability of non-malignant tumors of the womb and ovaries by medical treatment, and also to state the grounds on which it is founded. Regarding such growths as the product of a morbid or pathological condition, and believing in the possibility of correcting such condition by well directed treatment, I see no reason why the growth should not cease when thus deprived of its originating and sustaining cause ; nor why it should not only cease to grow, but on the contrary, gradually diminish from disintegration and absorption. This conviction, or opinion, is founded upon an event connected with the history of such growths, and which is known to the profession generally, namely, the spontaneous cures which occur now and then from the unaided efforts of nature ; and which, in my opinion, should inspire confidence in the use of remedies in aid of the good work. Notwithstanding the positive assertions to the contrary by many distinguished gentlemen, that there is no known remedy or treatment calculated to influence favorably such growths, or that can cause absorbtion of their contents, and accounting as they do, when such collections disappear, that the cyst ruptured, permitting the escape of the fluid into some other cavity or outlet, from which it is either absorbed or escapes. If such be the only mode or modes of its disappearance, I count myself most fortunate in the cases presented below for such a happening in so many instances. I am, however, fully persuaded as to the possibility of meeting the demands in a large proportion of cases, without a resort to surgical interference ; which, at best, is a humiliating confession as to the impotency of therapeutical appliances, from which every conscientious physician should strive to redeem his calling, as well as to save his patient from the additional hazard attending surgical relief; not because it is an untried expedient, but because of the unavoidable risk encountered under the most favorable circumstances, and in the hands of the most skillful operator. In making this statement, I would not have it understood as a reflection on the judgment or skill of the many distinguished gentlemen whose well-earned fame in uterine surgery has given them a world-wide reputation, nor do I mean to say there will no longer be an occasion for their services, but trust it may be but an occasional necessity, and not, as heretofore, the only hope. That the grounds of my belief are not irrational, must I think be admitted, not only from the facts above stated, but from the result in the following cases. Whether the result mentioned will ever be duplicated, either in my experience or others, remains a question for future solution, but let it be what it may, the impossibility theory or idea should find no place in medical parlance. Had such an humiliating conviction influenced our predecessors, many of the noblest triumphs of the past would have held no place in the list of medical achievements ; while, on the other hand, many opinions, once thought to be founded on the granite rock of truth, have, in consequence of enlightened progress and investigation, been disproved and abandoned. Others are gradually giving way to an advanced therapeutics. For instance, as to the best climatic conditions for the arrest and cure of consumption, the views, now rapidly gaining ground are diametrically opposed to those formerly entertained. To the spirit of impartial investigation and experience I commend this article, and if it should prove the inspiration by which one life is saved, I shall be amply repaid for the adverse criticism it is likely to invoke.

### OVARIAN TUMOR OF RIGHT SIDE.

The first case treated was in a colored woman, C. H., age about thirty years, mother of several children, but for some years past had been subjected to repeated abortions, and also menstrual irregularities. She had experienced at times a pain and dragging sensation in the right iliac region. Some months prior to her visit to my office she had noticed a lump in the right side, and which had gradually increased in size up to its present dimensions, which was about that of the fœtal head at full term. An examination satisfied me that the growth was an ovarian tumor, the product of inflammation of a Graafian follicle, and sustained by a continuance of the inflammatory action, which was evidenced by the tenderness and pain induced by handling the tumor. The patient presented evidences of impaired health, such as anæmia, a swollen condition of the lower extremities, constipation, and poor appetite. This being my first case, I entered upon the treatment with many misgivings, but determined to make an honest effort to suspend the morbid condition upon which I believed the growth to depend, and as far as possible bring every organ and function into a healthy condition and action. I gave on alternate nights one grain of calomel triturated with three grains of saach. alba. Gave twenty grains of muriate of ammonia in solution four times a day, and repeated blisters over the tumor till all tenderness or pain on handling had disappeared.

The improvement in the general health was soon manifest, and with this a gradual diminution in the volume of the tumor, which at the end of six months had entirely disappeared, and left the patient, to all appearances, in perfect health, and which, I am happy to say, has continued, as I am informed. During my acquaintance with her after the treatment she had two full-time, healthy children born, and I understand has had other healthy gestations since her removal to another State.

The second case was in a lady of some thirty-six years old, the mother of four healthy living children. She had for several years been troubled with uterine derangement of some sort, with impairment of general health. In course of time she noticed a hard lump in the left iliac region, which continued to enlarge until its true nature was determined, and pronounced by her attending physician an "ovarian tumor," for the removal of which he proposed an operation. This she declined. The tumor continued to grow until she presented the appearance of a lady at seventh month's gestation. At this time she sought the advice of a distinguished physician and surgeon, who confirmed the diagnosis, but thought the tumor too large in her debilitated condition to justify an operation, and gave some general directions for the management of her health, without making any special effort for the relief of the growth by medical treatment. A short while after this, in the fall of 1876, I was called to see her, and found her in a state of great debility, with a sallow, bloodless complexion, with feet and ankles bloated, bowels constipated, monthly periods irregular, sometimes scanty, at others profuse. The tumor reached to the umbilicus and was tender on being handled, especially so towards its neck or attachment. In this condition I first saw and prescribed for her. The improvement was prompt in both her general condition and also in the tumor. She remained under my immediate care for nearly three months, when the tumor had declined to about one-third of its original size. In color, complexion, flesh and spirits, as well as in appearance, she looked and acted like a well woman, with no appearance of any lump or inequality in her form. In this condition she returned home and resumed her profession of portrait painting, and feeling so well she discontinued the treatment, but has suffered no inconvenience since, notwithstanding the small remnant of the tumor remains, which she says she feels satisfied would have entirely disappeared had she continued the treatment. This case was treated in the fall of 1876.

Since writing the above I have had a communication from the lady in which she says: "I am in good health, and have never suffered a moment's discomfort from the tumor, and it is now scarcely discernible, and I find myself capable of discharging the duties appertaining to my business of landlady in an "Exposition boarding-house."

OVARIAN TUMOR.

The fourth case, T. R., a colored chambermaid in a hotel, age twenty-five, was treated in 1878. The tumor was of the right ovary, and appeared to the touch about the size of the fœtal head at six months inter-uterine life. She was unable to give the exact date of its appearance, but had for several years suffered at her menstrual periods, and for a couple of years before con sulting me had suffered at times with a "drawing pain," as she termed it, in the right ovarian region, and for several months had noticed a lump in that locality, which had gradually enlarged to its present size. She was unmarried and had never borne children. A few months' treatment dissipated the tumor and left her in good health, which has continued up to the present time.

Fifth case of inflammation and enlargement of the right ovary. The subject, Mrs. P., age twenty-six, mother of four living children; had suffered for over two years with pain in the right ovary. Had been treated previously by blisters and alteratives with benefit, but not relieved. When I examined her, I found a hardness in the ovarian region, quite tender to the touch. In this case I used a seton instead of blisters, and used the ammonia and calomel, as in the other cases, and in two months the trouble ceased with a disappearance of the hardness, since which time she has remained well. Treated in 1882.

The sixth case was in a colored woman, age thirty-five, mother of several children. Had suffered for some years with menstrual irregularities, attended by great pain at times and a profuse flow, compelling her to keep her bed for days at a time. The seat of the pain, she says, was in the right side, in which she further says she discovered a lump some months before consulting me. Upon examination I found a tumor about the size of a large apple, hard and quite tender on being pressed. The same course of treatment as before enumerated, in the course of six months, gave relief to all the evidences of disease, and so far as my knowledge goes, has continued well to the present time. Treated in 1882.

The third case, in the order in which they were treated, I have reserved for the conclusion, as it presents points of more than ordinary interest, in that it was diagnosed by two distinguished medical men prior to my first visit, as a fibro-cystic tumor of the womb, in which opinion I fully concurred on meeting them in consultation. The interest is heightened by the disappearance of a partially organized structure (or at least such we supposed it to be) under the combined operation of the alteratives and counter-irritation, and which I do not hesitate to say, was a result far beyond my most sanguine expectations.

FIBRO-CYSTIC TUMOR OF WOMB.

Mrs. W., the subject of this growth, was forty-four years old at the time I first saw her, and had been an invalid, as her husband writes me in a recent letter, since 1869, with symptoms pointing to the womb attended by pain, tenderness, and a profuse leucorrhœal discharge, and for which she was treated, but without affording relief. Menstrual irregularities accompanied her other troubles. Her health continued to decline, the discharge from the womb assuming more of a purulent character and an offensive odor. Ill health continued on for two or three years, when she noticed an enlargement of the abdomen, which was mistaken for pregnancy. When convinced of their error, the family physician was called in and made a careful examination and pronounced the enlargement due to a tumor of the womb, which he says in course of time enlarged, at the same time her general health continued to give way. The tumor, he says, at that time was about the size of a small child's head; which so changed her appearance as to preclude her leaving home had she been otherwise able. She was, however, so debilitated as to render her unfit for any household duties. She was seen by a distinguished physician and surgeon in connection with his family physician. Their united opinion was that the growth was a fibro-cystic tumor of the womb, and that it did not admit of surgical interference with a view to its removal. Soon after this consultation he writes: "Having heard of some such case treated by yourself, and learning that the patient was then sojourning in our city, I procured a letter of introduction from you and made her acquaintance (the case referred to is No. 2), and learned from her the result in her case, which greatly encouraged me, and induced me to request my family physician to call you in consultation, and also the gentleman mentioned in former consultation. I was informed you all agreed as to the character of the tumor, "fibro-cystic of the womb," and that it was not a case for surgical interference."

The letter goes on to say: "My wife took your treatment for four or six months, when we considered her well, and that she has continued to enjoy good health up to the present time, the tumor having entirely disappeared, so that it cannot be felt." He also says she has passed the menstrual climax without interruption to her health, and that she is now fifty-three years old, but with a cheerfulness and healthy elasticity of one at thirty. It is now eight years since she abandoned her treatment, which I think gives a guarantee of the permanency of the cure.

I have thus let the husband speak of the past and present condition of his wife's health, to show that even under the dicouraging opinion we held as to the nature of the growth, the success has been most gratifying and, to my mind, most encouraging. I will only add that the same course of treatment was pursued in this as in the other cases.

